# Clinical mentorship of midwifery students: The perceptions of registered midwives

**DOI:** 10.4102/hsag.v29i0.2492

**Published:** 2024-04-30

**Authors:** Hafaza B. Amod, Lindani Ndlovu, Petra Brysiewicz

**Affiliations:** 1 Department of Nursing, Faculty of Nursing and Public Health, University of KwaZulu-Natal, Durban, South Africa; 2 Department of Maternity, Department of Health, Durban, South Africa

**Keywords:** clinical mentorship, midwifery students, registered midwife, qualitative research, South Africa, clinical support

## Abstract

**Background:**

Clinical mentors are experienced practitioners who play an important role in encouraging the professional development of students in clinical areas. The responsibility of clinical mentorship in nursing is often difficult to maintain. However, there is a dire need for clinical mentorship in maternity units, especially in South African hospitals were high maternal mortality rates remain alarmingly high.

**Aim:**

This study aimed to describe the perceptions of registered midwives regarding the clinical mentorship of midwifery students.

**Setting:**

The study occurred in a semi-rural state regional hospital in the eThekwini district, KwaZulu-Natal.

**Methods:**

A qualitative exploratory and descriptive design was conducted using in-depth individual interviews with midwives in maternity units. A purposive and convenient sampling method recruited 17 registered midwives from 3 maternity care areas within a single setting. Interviews were audio-recorded and all data were analysed using qualitative content analysis.

**Results:**

Five categories emanated from this study namely, sharing knowledge and skills; encouraging role model behaviour; promoting self-worth; Is a challenging task; and requiring additional support.

**Conclusion:**

Clinical mentorship has a reciprocal effect on teaching and learning in maternity care areas and encouraged registered midwives to lead as role-models. The process demands competence, professionalism, and leading by example. Despite the confidence, satisfaction and interest in clinical mentorship, registered midwives often find the process challenged by patient care priorities. Therefore, registered midwives require additional support to mentor students in clinical practice.

**Contribution:**

This article shows that clinical mentorship places various challenges on registered midwives and formal mentorship training could be beneficial.

## Background

Mentorship is a nurturing relationship in which a more experienced or knowledgeable practitioner (mentor and/or role model) guides a less experienced or less knowledgeable practitioner, known as the mentee (Leonard, McCutcheon & Rogers 2016). In nursing, clinical mentoring is defined as a collaborative relationship between an experienced nurse and a student in the clinical area where the relationship is based on improving the personal and professional growth of the novice nurse (the student) (Zhang et al. 2016).

Clinical mentorship in nursing is extremely crucial during student placement in clinical learning environments as it allows students to practice the skills required to become competent professional nurses (Foolchand & Maritz 2020). According to Tuomikoski et al. (2019), mentors play an important role in encouraging the professional development of students, and students regard clinical mentors as vital role models. Dobrowolska et al. (2016) suggest that mentorship assists students in bridging the theory–practice gap and developing critical skills, which in turn enhances students’ clinical learning experiences. The function of a clinical mentor includes providing support and guidance to facilitate clinical learning, partake in clinical assessment, and ensure that mentees are well-equipped for practice (Salifu 2016). However, Herron et al. (2016) and Setati and Nkosi (2017) reported that registered nurses and midwives often encounter a role conflict in practice between providing patient care and clinical support responsibilities. Challenges such as a lack of resources, high patient turnovers, and high student intakes contribute to increased workload responsibilities (Thopola 2015). According to Lescano et al. (2019), clinical mentorship is not commonly practised in resource-limited countries.

In South Africa, clinical mentorship in healthcare facilities is not clearly developed and often occurs informally and on a voluntary capacity (Amod & Mkhize 2022). The lack of support and training for qualified practitioners is likely associated with the omission of mentorship as a clinical responsibility (Mikkonen et al. 2022). More recently, during the coronavirus disease (COVD-19) pandemic, the urgency to save lives surpassed other clinical responsibilities, including mentorship. Fewer learning opportunities left students feeling lost in a chaotic environment (Ulenaers et al. 2021; Voigt et al. 2022), and frustrated that no guidance was available in the student’s time of need (Dziurka et al. 2022). Hence, there is a strong demand for good clinical role models to positively impact students’ confidence and professional development. Clinical mentorship is considered an important professional development strategy for improving clinical performance and outcomes (Kung et al. 2023).

In maternity departments, midwives are often expected to care for the pregnant woman and foetus, manage the birthing process, and ensure healthy postdelivery outcomes for both the mother and the baby. The double responsibility placed on midwives demand that they remain highly competent to achieve good maternal and neonatal health outcomes. With such demands, a supportive and caring role of a mentor is likely to encourage midwives to continue providing high quality of nursing care (Anderson et al. 2022). Currently, the South African healthcare system is overburdened with numerous litigation, specifically in maternity services (Mathope, Du Preez & Scheepers 2023) where unskilled practitioners are often cited as a contributing factor to poor maternal health outcomes (Greenway, Butt & Walthall 2019; Mathope et al. 2023). A study by Wissemann et al. (2022) promotes mentorship as a means to retain staff and improve clinical and organisational outcomes. Similarly, clinical mentorship of midwifery students, who are the future of the profession, is extremely important as the authors work towards improving maternity care outcomes.

The clinical mentorship experiences of midwifery students have been described; however, little is known about the clinical mentorship experiences of their mentors (the registered midwives) in a South African setting.

## Purpose of the study

To describe the perceptions of registered midwives regarding clinical mentorship of midwifery students in a local hospital in KwaZulu-Natal, South Africa.

## Research methods and design

A qualitative, exploratory and descriptive research design was adopted in this study.

### Research setting

The research setting was the midwifery departments of a large regional hospital within the eThekwini district of KwaZulu-Natal, South Africa. The hospital provides health services to the surrounding community, covering a population size of approximately 1.6 million residents. Midwifery students from a local university are placed at the facility for their clinical practice. The maternity department in this hospital is divided into three units: the antenatal unit, the intrapartum unit as well as a postnatal unit. The antenatal unit is made of a single antenatal clinic and two antenatal wards. Each ward has 40 beds each. The intrapartum unit consists of an admission room, labour ward, and maternity high care unit. The labour ward has 15 beds and the maternity high care unit consists of 5 beds. The postnatal unit is made of two 40-bedded post caesarean section units, and two 40-bedded postnormal vaginal delivery units. There are approximately 6 qualified midwives with 6–8 students who are placed in each unit. However, the number of students is also dependent on the students clinical and block timetables.

### Research participants

The researcher used purposive sampling technique to recruit registered midwives from the maternity departments and sample size was determined once data redundancy was reached; a decision made in consultation with the entire research team where one member is an experienced qualitative researcher.

#### Inclusion criteria

Nurses with midwifery qualification working in maternity wards and registered as a midwife with the South African Nursing Council.

#### Exclusion criteria

Registered midwives undergoing community service; registered nurses who are students placed for clinical learning; and registered nurses who are not permanently employed at the hospital were excluded from participation in this study.

### Data collection process

The data collection process commenced immediately after obtaining ethical approval from the affiliated university and the Department of Health ethics committee, as well as permission from the hospital. The researcher (L.N.), who was currently employed at the local hospital, met with the assistant nurse manager to discuss the purpose of the research and the data collection process. The researcher was then invited to brief potential participants at a department meeting, where she presented the research purpose and the data collection process to registered midwives working in the department. The researcher scheduled appointments with interested individuals according to their availability, thus ensuring it did not interfere with their duty schedules, as agreed with the nurse manager. An information sheet and a consent form were distributed to each participant and collected prior to the commencement of the interview. All individual interviews were conducted in the maternity department lecture room with the permission of the assistant nursing manager. The researcher used a semi-structured interview guide to lead the interview, and used probing questions to gain more information where necessary. The interviews lasted approximately 40 min – 50 min each, and data collection took place over 3 weeks (February to March 2020). A total of 17 interviews were completed. The research team determined that data redundancy was reached after 15 participants as the interviews were generating similar information. The decision was then made to do an additional two interviews and to stop data collection.

### Data analysis

Qualitative content analysis outlined by Erlingsson and Brysiewicz (2017) guided the data analysis and the steps included:

- Getting acquainted with the data by reading and re-reading the transcribed interviews several times to gain an understanding of the participants’ responses
- The text was divided into meaning units and condensed further (while ensuring core meanings were retained)
- The condensed meaning units were then coded
- Codes were then grouped into categories

### Ethical considerations

The principles of respect, confidentiality, autonomy and non-maleficence was maintained in the study. Gatekeeper approvals from the National Department of Health-South Africa (KZ 201811029), the Chief Executive Officer of the research setting (56/RESH/2018). This study has been ethically reviewed and approved by the UKZN Humanities and Social Sciences Research Ethics Committee (Approval Number: HSS/1845/018M). All participants provided written consent to participate and use audio recording during the interview. Confidentiality was practised throughout the study. Participants were free to withdraw from the study at any stage of the data collection process. The study did not involve any harmful practice. All data collected were kept secure and only accessible to the researcher and her supervisors.

### Trustworthiness

*Credibility*: The researcher was familiar with the culture of participants at the selected setting as she is employed in the same hospital. A prolonged relationship and good rapport facilitated a relaxed and trusting environment between the researcher and the research participants. Only participants willing to partake in the study were included, encouraging them to speak freely about their clinical mentorship experiences. Participants were reminded of their right to withdraw from the study at any given time without any repercussions. During the interview, the researcher used probing to clarify and understand what participants were saying, which produced detailed and accurate information about their experiences. Data analysis and interpretation of findings were reached and agreed upon after frequent meetings between the researcher and researcher supervisors to prevent researcher bias and incongruencies in data.

*Transferability*: The researcher provided detailed descriptions of the research setting and process, as well as information about clinical mentorship within the eThekwini district. The information provided attempts to demonstrate that the knowledge acquired may be relevant to a similar situation or participants.

*Confirmability*: A step-by-step process of data collection, analysis and interpretation was undertaken. All discussions, procedures and decisions were conducted with two research supervisors, and an audit trail for future tracking was saved electronically.

*Dependability*: A detailed description of the research methodology was provided in a detailed report to allow future researchers to repeat the study within a similar context (Lincoln & Guba 1985; Shenton 2004).

## Results

### Demographic profile

There were 17 participants in this study. Six participants were registered midwives who completed an undergraduate degree (*n* = 1) or diploma (*n* = 5) programme in Nursing. Eleven participants were clinical specialist who completed a postgraduate qualification, which was either a master’s degree in maternal and child health or an advanced diploma in midwifery course. Table 1 displays the participants’ profiles.

**TABLE 1:** Demographic profile of the participants.

Participant number	Level of qualification	Current position	Years of experience
1	4-year diploma in nursing	Registered nurse and midwife	08
2	4-year diploma in nursing	Registered nurse and midwife	20
3	4-year diploma in nursing	Registered nurse and midwife	09
4	4-year diploma in nursing	Registered nurse and midwife	05
5	4-year diploma in nursing	Registered nurse and midwife	9
6	4-year degree in nursing	Registered nurse and midwife	12
7	Advanced diploma in nursing	Clinical specialist (advanced midwifery)	10
8	Advanced diploma in nursing	Clinical specialist (advanced midwifery)	15
9	Advanced diploma in nursing	Clinical specialist (advanced midwifery)	20
10	Advanced diploma in nursing	Clinical specialist (advanced midwifery)	23
11	Advanced diploma in nursing	Clinical specialist (advanced midwifery)	21
12	Advanced diploma in nursing	Clinical specialist (advanced midwifery)	06
13	Advanced diploma in nursing	Clinical specialist (advanced midwifery)	14
14	Advanced diploma in nursing	Clinical specialist (advanced midwifery)	18
15	Master’s degree in nursing	Clinical specialist (advanced midwifery)	15
16	Master’s degree in nursing	Clinical specialist (advanced midwifery)	10
17	Master’s degree in nursing	Clinical specialist (advanced midwifery)	19

### Main findings

Five categories emerged from the data, namely sharing of knowledge and skills; encouraging role model behaviour; promoting self-worth; it’s a challenging task; and requiring additional support.

#### Sharing of knowledge and skills

Registered midwives viewed clinical mentorship as sharing of knowledge and the transferring of skills to correlate theoretical information with practical experiences. Thus, clinical mentorship was seen by the participants as preparing students to be competent and independent in performing clinical tasks. Participant 4 described that clinical mentorship:

‘[Is] having knowledge and giving knowledge to someone who has lesser knowledge than you. So, you impart knowledge to someone to do as you do or better than you.’ (P4, female, Registered midwife)

Another said:

‘It’s [*mentorship*] a good thing, because you must know nursing by the tip of your fingers, so you understand it because you need to pass on this knowledge to others.’ (P7, female, Midwifery clinical specialist)

Another participant described this sharing of knowledge as being:

‘… when a senior or knowledgeable or skilled professional is helping the learners in terms of practical skills that are conducted in maternity.’ (P14, female, midwifery clinical specialist)

Participant 3 added:

‘Teaching them [*students*] the practical skills required [*them*] to put theory into practice.’ (P3, female, registered midwife)

Findings revealed that the sharing of knowledge and transferring of skills during clinical mentorship was beneficial to students and the future of the profession. Participant 6 explained:

‘A well-mentored student is a productive student. That student can perform his or her duties in isolation [*independently*] without supervision. Students learn to be independent because that student has a clear understanding of what to do.’ (P6, female, registered midwife)

Participant 17 added:

‘So, to mentor a student is just an investment. Indirectly you’re just planting the information to somebody who will be carrying on the profession.’ (P17, female, midwifery clinical specialist)

Sharing of knowledge and skills with students is important when preparing them for competence and independent role-taking. The future of the profession lies in the hands of students who are currently in training. Thus, clinical mentorship is an investment in the profession.

#### Encouraging role model behaviour

Participants found that when students are placed for clinical learning and practice, registered midwives are expected to be competent, display professionalism, and lead by example because students learn from what they see. Therefore, it is important that registered midwives project the qualities of a good role-model while preparing students for role-taking.

Participant 12 explained:

‘You become sort of their role model so that they can copy what you do, if there’s questions you answer and lead them in the right path in the profession.’ (P12, female, midwifery clinical specialist)

Other participants supported this by saying:

‘To mentor someone is to set an example for that person. So, she has to follow your steps and to look at your actions how you do your job, your behaviour, for example punctuality …’ (P13, female, midwifery clinical specialist)

Being competent, professional and leading by example are some of the characteristics of a good role-model. The study participants found that clinical mentorship encouraged them to adopt such behaviour.

#### Promoting self-worth

Some registered midwives indicated that clinical mentorship assisted them to refresh their midwifery knowledge to facilitate the teaching of midwifery students during clinical practice. This afforded them an opportunity to reflect on their own practices and encouraged learning for teaching. Participant 10 said:

‘For me, I also benefit because it makes me to revise things. To refresh my mind, I have to think about it, and if it’s something that doesn’t usually happen, I go back to my books to check on it so that I give the correct information. So, it keeps me updated.’ (P10, female, midwifery clinical specialist)

Participant 13 mentioned:

‘Okay, first of all as the mentors of students, you also gain the knowledge. You go out and study more and come back and give the students more knowledge … It’s like we starting afresh from giving them new knowledge- the practical part of it.’ (P13, female, midwifery clinical specialist)

While registered midwives found clinical mentorship to be interesting, imparting knowledge to midwifery students provided a sense of inner satisfaction and confidence. This was emphasised in the following excerpts:

‘Firstly, I would start by myself, I benefit too much because I become confident that I can give someone some ideas.’ (P13, female, midwifery clinical specialist)

Participant 9 stated:

‘It’s an interesting thing to help somebody to gain more knowledge and have confidence on whatever she or he is doing and to make that person confident enough to do that thing on his or her own. And that gives me satisfaction …’ (P9, female, midwifery clinical specialist)

She added on by saying:

‘You teach that person how to do an episiotomy and if you give that person an opportunity, the next time the person will get better with explanation and understanding until they can do it on their own. And that gives me satisfaction that this person was not having any information on how to do it [*episiotomy*] and now the person is confident in doing it. It gives me satisfaction. So, I think mentoring is a good thing.’ (P9, female, midwifery clinical specialist)

These remarks from participants showed that while the process of clinical mentorship enhanced the mentees’ level of confidence and independence, it also assisted them in gaining satisfaction from relaying knowledge and skills. Hence, clinical mentorship contributed to improved self-worth in both registered midwives and midwifery students.

#### It’s a challenging task

Some registered midwives working in the busy labour ward perceived clinical mentorship to be a difficult and time-consuming activity. They found it challenging, especially during times when patient turnover and staff shortage were of concern. The negative attitude of some students was another factor that contributed to the challenge of clinical mentorship.

One participant said:

‘To be honest, with me it’s very difficult. I would say I don’t like to do it in a way that it takes up a lot of time. We don’t have time, especially in this department in this labour ward.’ (P15, female, midwifery clinical specialist)

Another participant added:

‘At times like currently, it [*clinical mentorship*] is kind of impossible because we are just faced with this situation like a real shortage [*of staff*] and there is an overflow of patients. We are not coping, we are reaching burnout, we are overworked. So, by the time you need to teach this person because they are coming here to gain knowledge, but you usually don’t have the time.’ (P3, female, registered midwife)

Participant 8 explained:

‘… its time consuming, so there should be a specific individual that is solely for mentoring … Let’s say you were demonstrating an episiotomy, you need to talk about infiltration and also the proper cutting of the episiotomy and then the suturing of the episiotomy in layers. But when you’re doing it yourself, you find that the procedure will take 10 minutes but it takes up to an hour when you’re demonstrating it to a student.’ (P8, female, midwifery clinical specialist)

Participant 10 complained about students’ attitudes toward clinical learning. She said:

‘Some of the students are not serious about their work and they don’t even approach you for help … if someone isn’t like eager to learn, it discourages you. So, in other words it depends on their attitude.’ (P10, female, midwifery clinical specialist)

Findings revealed that the practice of clinical mentorship is highly reliant on the current clinical situation and the attitude of students placed for clinical learning. High patient turnovers, staff shortages, and negative student attitudes are challenges for clinical mentorship.

#### Requiring additional support

Some participants felt that they required additional support to conduct effective clinical mentorship. They expressed that lecturers of nursing education institutions often place midwifery students in the units without providing clear expectations on clinical learning goals. One participant verbalised:

‘So, we don’t know what they have learned or what they have not learned.’ (P4, female, registered midwife)

A second participant expressed:

‘Sometimes it’s like they have been just dumped there! There is no one who bothered to come or communicate with the unit.’ (P1, female, registered midwife)

The lack of communication regarding clinical mentorship expectations from nursing education institutions was a major concern. One participant expressed:

‘I don’t see much of them [*tutors from colleges*] here, so I think if maybe possible if they can come few times to orientate and show the students first.’ (P7, female, midwifery clinical specialist)

Another added:

‘We would like to communicate regarding what we expect of these students when they come out, or this is what we have done as far as the practical [*requirements*] are concerned.’ (P5, female, registered midwife)

Registered midwives felt that better communication processes with lecturers regarding students’ expectations and mentorship responsibilities would enable them to conduct more effective clinical mentorship experiences.

## Discussion

In keeping with the views of registered midwives from this study, Moked and Drach-Zahavy (2016) agree that clinical mentorship is the sharing of knowledge and the transferring of skills to prepare midwifery students towards clinical competence and independence. The same was reiterated by McIntosh, Gidman and Smith (2014), who also showed that by sharing their knowledge and skills, registered midwives acquaint themselves with clinical mentorship role expectations, thus creating positive mentor roles that significantly contribute to the retention of students in the nursing profession (Salifu 2016; Thunes & Sekse 2015; Tuomikoski et al. 2019). In this study, registered midwives shared similar thoughts and insinuated that competent and independent practitioners are produced through clinical mentorship, thus securing the future of the profession. Hence, clinical mentorship is an investment in the nursing profession.

Role modelling positively impacts students learning while in clinical placement (Van Graan, Williams & Koen 2016). This study’s findings showed that clinical mentorship encouraged registered midwives to adopt role-model behaviour. Registered midwives updated themselves with recent knowledge and skills and displayed professional behaviour so that they could lead by being a good example for students to follow. This effect of learning for teaching promoted high expectations of the role. Reflecting on their clinical mentorship roles left registered midwives feeling satisfied because many competent and independent practitioners were produced through their guidance. Thus, promoting a sense of self-worth.

According to McInnes et al. (2015), Malwela, Maputle and Lebese (2016) and Bowen, Kable and Keatinge (2019), registered nurses who are overburdened with patient care duties often negated student supervision opportunities. Similarly, this study’s findings indicated that registered midwives were challenged with clinical demands related to high patient turnovers, shortage of staff and time constraints. Additional support from lecturer and improved communication about clinical mentorship role expectations and responsibilities would improve the quality of clinical experiences. Similarly, Phuma-Ngaiyaye et al. (2017) and De Swardt et al. (2017) highlighted that poor communication between educational and clinical settings, is a global challenge in the nursing profession.

There is a growing need for registered midwives to assume their roles as mentors to students placed for clinical learning in maternity care areas. Phuma-Ngaiyaye et al. (2017) point out that a potential cause of theory–practice gap could be a lack of training in mentorship, which often leads to feelings of unpreparedness to take on the clinical teaching role.

### Limitations

This research study was conducted at a single state regional hospital located in eThekwini district in KwaZulu-Natal and therefore, the results may differ in other contexts.

Moreover, the participants of this study were known to the researcher and this may have influenced participant responses.

### Recommendations

Improved communication between lecturers from nursing education institutions and registered midwives will likely improve clinical mentorship roles and responsibilities. Registered midwives in South Africa undertake clinical mentorship of students in a voluntary and informal capacity. There is insufficient priority given to clinical mentorship and therefore, registered midwives need additional support and formal mentorship training to adequately mentor students during clinical learning.

## Conclusion

Clinical mentorship in nursing is a process of supporting students by sharing knowledge and skills to correlate theory with practical experiences. Hence, clinical mentorship prepares students to be competent and independent in performing clinical skills needed for role-taking, thus investing in the profession’s future. Registered midwives found that clinical mentorship encouraged them to act as role-models so that students may imitate and adopt similar traits. Clinical mentorship showed benefits that promoted the self-worth of registered midwives and students. Despite the confidence, satisfaction and interest in clinical mentorship, registered midwives often find the process challenged by time constraints, patient care priorities, staff shortages, and negative student behaviours. Therefore, training in mentorship will likely provide the support needed to undertake clinical mentorship, more effectively.
